# Caring for Persons With Dementia in Audiology

**DOI:** 10.1093/geroni/igab046.327

**Published:** 2021-12-17

**Authors:** Marilyn Reed

**Affiliations:** Baycrest Health Sciences, Toronto, Ontario, Canada

## Abstract

While hearing loss is highly prevalent among patients with dementia, it frequently goes unidentified and unmanaged. It has been a commonly-held belief that older adults with dementia are unable to benefit from hearing rehabilitation, but recent evidence shows that many individuals with dementia can successfully use amplification, helping to improve communication, social interaction and quality of life for these individuals and their caregivers. This presentation will describe how modifications to practice led to successful outcomes for the majority of patients of a geriatric audiology clinic with co-morbid hearing loss and cognitive impairment. In a study of hearing aid use in 67 patients with these comorbidities, over 90% used hearing aids successfully with measurable benefit for both patients and caregivers. Furthermore, we will discuss approaches to improving communication for LTC residents with dementia and hearing loss through the support of audiologists during remote visits with physicians and families during the pandemic.

